# Targeting 3D chromosomal architecture at the RANK loci to suppress myeloma-driven osteoclastogenesis

**DOI:** 10.1080/2162402X.2022.2104070

**Published:** 2022-08-01

**Authors:** Katja Thümmler, Mark TS Williams, Susan Kitson, Shatakshi Sood, Moeed Akbar, John J Cole, Ewan Hunter, Richard Soutar, Carl S Goodyear

**Affiliations:** a Institute of Infection, Immunity and Inflammation, College of Medical, Veterinary and Life Sciences, University of Glasgow, Glasgow, UK; b GLAZgo Discovery Centre, Institute of Infection, Immunity and Inflammation, College of Medical, Veterinary and Life Sciences, University of Glasgow, Glasgow, UK; c Oxford BioDynamics, Oxford, UK; d Beatson West of Scotland Cancer Centre, Gartnavel Hospital, Glasgow, UK

**Keywords:** FcγR, epigenetic, RANK, multiple myeloma, osteoclasts

## Abstract

Bone disease represents a major cause of morbidity and mortality in Multiple Myeloma (MM); primarily driven by osteoclasts whose differentiation is dependent on expression of RANKL by MM cells. Notably, costimulation by ITAM containing receptors (i.e., FcγR) can also play a crucial role in osteoclast differentiation. Modeling the pathology of the bone marrow microenvironment with an *ex vivo* culture system of primary human multiple myeloma cells, we herein demonstrate that FcγR-mediated signaling, via staphylococcal protein A (SpA) IgG immune-complexes, can act as a critical negative regulator of MM-driven osteoclast differentiation. Interrogation of the mode-of-action revealed that FcγR-mediated signaling causes epigenetic modulation of chromosomal 3D architecture at the RANK promoter; with altered spatial orientation of a proximal super enhancer. Combined this leads to substantial down-regulation of RANK at a transcript, protein, and functional level. These observations shed light on a novel mechanism regulating RANK expression and provide a rationale for targeting FcγR-signaling for the amelioration of osteolytic bone pathology in disease.

## Introduction

Under normal physiological conditions bone remodeling, driven by osteoclasts (OC) resorbing bone and osteoblasts (OB) forming new bone, is tightly regulated. Various diseases, such as rheumatoid arthritis, primary bone tumors, and multiple myeloma (MM), are however characterized by an imbalance in normal bone remodeling with an increase in osteoclast activity and decreased osteoblast differentiation and survival. OC differentiation (osteoclastogenesis), from myeloid precursors, is driven by two main cytokines; macrophage colony-stimulating factor (M-CSF) and receptor activator of NFκ-B ligand (RANKL).^1^ Expression of RANK, the corresponding receptor for RANKL, is essential for osteoclastogenesis, with *RANK* ^/-^ mice exhibiting profound osteopetrosis, resulting from a complete lack of OCs.^2^

Multiple Myeloma, which is the second most common hematologic malignancy, is a neoplastic plasma cell disorder characterized by the clonal expansion of malignant plasma cells in the bone marrow. A hallmark of the disease is bone destruction in the form of osteolytic lesions causing severe bone pain, fractures, osteoporosis, and spinal cord compressions. Many mechanisms have been postulated to explain the enhanced osteoclastogenesis observed in MM, including raised levels of RANKL, chemokines (i.e., CCL3), pro-inflammatory cytokines, and cell–cell contact signaling. Molecular cross-talk between RANKL present on, and secreted by, MM plasma cells and RANK on osteoclast precursors (OCPs) represents a key mechanism driving osteoclastogenesis and subsequent bone pathology in MM.^3^ In turn OC secreted factors, such as IL-6 and BAFF/APRIL, in conjunction with cell–cell contacts induce the proliferation and survival of MM plasma cells, establishing a vicious cycle of tumor survival and bone erosion.^[4,5]^ In order to find strategies to interfere with this ongoing pathology in the bone marrow microenvironment, it is crucial to understand what drives this inter-dependence of lytic bone disease and myeloma growth.

Importantly, in addition to the RANKL/RANK interaction, other immunoreceptors are critical in providing costimulatory signals for osteoclastogenesis including the immunoreceptor tyrosine-based activation motif (ITAM) containing receptors DAP12 and FcR.^6,7^ We and others have previously demonstrated Fc**γ**receptor (Fc**γ**R) expression on (pre)osteoclasts,^8–12^ and investigated the functional consequence of Fc**γ**R engagement by immunoglobulins and immune complexes (IC). Notably, depending on the composition of the IC, they can either enhance or inhibit osteoclastogenesis. In general, antigen-specific ICs and heat-aggregated IgG are associated with enhancing osteoclastogenesis^13,14^ whilst alternative IC preparation, such as IVIg^15^ or staphylococcal protein A (SpA) IgG small hexameric IC (SIC),^8^ can inhibit RANKL-driven osteoclastogenesis.

In this study, we investigated Fc**γ**R-mediated regulation of steady-state (RANKL) and malignancy (MM)-driven osteoclastogenesis. We also assessed whether this approach can inhibit OC-driven bone disease in MM. We used primary myeloma cells from active MM patients and primary human monocytes/pre-osteoclasts to establish a co-culture system mimicking the pathological conditions of the bone marrow microenvironment in MM. This complex *ex vivo* culture system enabled us to model the non-inflammatory osteolytic bone disease, as well as the enhanced tumor cell survival caused by excessive osteoclastogenesis. Herein, we demonstrate that Fc**γ**R targeting by SIC significantly suppresses RANKL and MM-induced osteoclastogenesis, and furthermore reduced myeloma cell survival. On a mechanistic basis, we were able to demonstrate that this correlates with the epigenetic modulation of the *RANK* promoter, and a marked reduction in RANK expression at the gene, protein, and functional level. Therefore, we have elucidated a novel mechanism regulating RANKL- and MM-driven osteoclastogenesis, and provide evidence suggesting that Fc**γ**R targeting may represent an attractive therapeutic strategy targeting bone disease and tumor burden in MM.

## Materials and methods

### Immune complex (SIC) and OVA-IgG (OpIgG) generation

Both SIC and OpIgG were generated as previously described by.^8^ Briefly, SICs were generated by incubating 37.5 µM SpA (rSPA, RepliGen) with 150 µM human IgG (Jackson Immunochemicals) in PBS at 37°C for 1 h. The OpIgG control (OVA plus IgG) was made by incubating 37.5 µM of OVA (Sigma) with 150 µM human IgG at 37°C for 1 h. To remove large pre-formed IgG complexes the IgG was centrifuged at 13,000 revolutions per minute prior to being used.

### Cell culture

Buffy coats of healthy human blood were obtained from the Scottish Blood Transfusion Service and fresh blood was received from multiple myeloma patients (use was approved by the Glasgow East Ethics Committee). Peripheral blood mononuclear cells were separated by density centrifugation over a Histopaque (Sigma-Aldrich) gradient, and monocytes were isolated using the CD14-positive selection EasySep kit (StemCell Technologies). Purity of CD14+ monocytes was analyzed by flow cytometry using the cell markers CD3, CD14, and CD19 (BD Biosciences). 1 × 10^6^ monocytes/ml were cultured in complete α-minimum essential medium (10% fetal bovine serum, 2 mM L-glutamine, 100 µg/ml penicillin, 100 µg/ml streptomycin; all Sigma) with 25 ng/ml human M-CSF (PeproTech) and after 12 hours 100 ng/ml human soluble RANKL (PeproTec) was added to generate pre-osteoclasts (2 days in culture) or osteoclasts (7 days in culture). Human IgG (150 μM), SpA (37.5 μM), SIC or OpIgG was added at the same time as the RANKL. Each culture condition was set up in triplicate, incubated at 37°C, 5% CO_2_ and media was changed every third day.

The human myeloma cell line U266B1 (ATCC) was cultured in complete RPMI 1640 supplemented with 15% heat-inactivated FBS, 100 µg/ml penicillin, 100 µg/ml streptomycin, 10 mM HEPES, 2 mM L-glutamine and 1 mM Sodiumpyruvate (all Sigma).

Following informed consent, diagnostic bone marrow samples from adults with active Multiple Myeloma were purified using the CD138-positive selection EasySep kit (StemCell Technologies) by positive magnetic selection according to manufacturer’s instructions. Purity of CD138+ plasma cells was routinely assessed by FACS (staining for CD45, CD138, and CD38, all from BD Biosciences). Myeloma plasma cells were maintained in complete DMEM; 20% heat-inactivated FBS, 100 µg/ml Penicillin/streptomycin and 2 mM L-Glutamine, with additional supplements; 1% MEM non-essential amino acids and 0.1% 2-mercaptoethanol (both from Gibco). Use of all human bone marrow samples was approved by the West of Scotland Research Ethics Committee. Myeloma cells were then used to drive osteoclastogenesis from monocytes or pre-osteoclasts in a coculture system in the absence of exogenous RANKL.^16^ To analyze osteoclast numbers, primary myeloma cells (1 × 10^6^/ml) or U266B1 cells (0.25 × 10^6^/ml) were co-cultured with pre-osteoclasts (1 × 10^6^/ml) for 7 days. For myeloma cell survival analysis, the primary or cell line myeloma cells were co-cultured with monocytes after overnight incubation with M-CSF (25 ng/ml), at the same densities for 3 days. In both cases, cells were co-cultured in the presence or absence of SIC, SpA, IgG, or OpIgG (added at the same time as the myeloma cells) with media changes every third day.

### Flow cytometry

Cells (0.5–1.0 × 10^6^) were stained with specific antibodies or isotype controls and in some cases pre-incubated with Fc Block. For the RANK surface staining, recombinant RANKL was fluorochrome-labeled (Pacific Blue Monoclonal Antibody Labeling Kit, Invitrogen) and incubated to the cells for 1 h at 37°C in 10% FBS-MEM α medium. For analyzing apoptosis rates, the cells were resuspended in Annexin V Binding Buffer (Miltenyi) and incubated with 5 µL AnnexinV-APC conjugate for 30 min at 4°C followed by staining with 7AAD. Data were acquired on a MACSQuant flow cytometer (Miltenyi Biotec) and analyzed with FlowJo7.2.4 software (Tree Star Inc).

### TRAP staining and osteolytic potential

Cells were stained for tartrate-resistant acid phosphatase (TRAP) using a leukocyte acid phosphatase kit (Sigma-Aldrich) according to manufacturer’s instructions. Osteoclasts were identified by light microscopy as TRAP^+^ (purple staining) large, multinucleated (≥3 nuclei) cells (images were blinded prior to analysis). Osteolytic potential of the cells was analyzed by culturing monocytes on bone slices (Immunodiagnostic Systems) with 25 ng/ml human M-CSF and 100 ng/ml human soluble RANKL in complete α-minimum essential medium in the presence or absence of IgG, SIC, SSpA,or OpIgG for 16 days with media changes every third day, followed by staining with wheat germ agglutinin (WGA)-lectin peroxidase conjugate (Sigma) and developed with DAB (3,3’-diaminobenzidine tetrahydrochloride, DAKO). Brown erosion pits were identified under light microscopy and erosion area was analyzed with ImageJ 1.440 software (NHS).

### Microarray profiling

RNA was extracted from primary human monocytes, differentiated with M-CSF (25 ng/ml) over night, followed by incubation with 100 ng/ml RANKL in the presence or absence of SIC or the appropriate Isotype control (IgG or SpA) for 24 h, using the Qiagen miRNeasy kit according to manufacturer’s instructions. RNA quality and integrity was checked using the Agilent Bioanalyzer 6000 Nano LabChip platform. The total RNAs were amplified and biotinylated using Illumina TotalPrep RNA kit following hybridization by Whole-Genome Gene Expression Direct Hybridization Assay to Illumina Human HT-12 V4.0 beadchip using manufacturer’s protocols. The hybridized arrays were scanned on the Illumina BeadArray Reader and raw data was quantile normalized with Illumina GenomeStudio (V2011.1, Illumina Inc.). Control (M-CSF/RANKL) and Treatment/Isotype Control (SIC, SpA, or IgG) groups were generated with three independent biological replicates per group (paired samples from three different donors) and the data has been deposited in the Gene Expression Omnibus database (https://www.ncbi.nlm.nih.gov/geo/query/acc.cgi?acc=GSE133210). The quantile normalized data was then analyzed with Partek Genomics Suite (version 6.6, Partek Inc.) software. Probe set level data was log2 transformed and transcripts were filtered for a low-expression cutoff (transcript expression below 6.1 in either control or treatment groups), resulting in 19,935 of 47,322 transcripts passing the threshold filter. Differential expression analysis of these 19,936 transcripts was then carried out by performing a paired ANOVA on the normalized expression values.

For genes with multiple probe-sets the probe-set with the lowest p-value was retained. The expression data was explored and visualized using Searchlight 2 (v1.0).^17^ Specifying all combinations of differential expression and using the Gene Ontology database Biological Functions (http://geneontology.org/). Gene ontology enrichment (for all up-regulated genes) was calculated using a hypergeometric test with BH correction and a significance threshold of adjusted p < .05. Principal Component Analysis was performed using all expression values and used per gene z-scores to reduce high-expression bias. The heatmap was generated using per gene expression z-scores and was clustered using Spearman distances, mean agglomeration, and re-ordering.

### PCR

Total RNA was extracted from cell lysates using RNeasy Mini kit (Qiagen) according to manufacturer’s instructions. RNA (100 ng) was reverse-transcribed using the Qiagen QuantiTect Reverse Transcription Kit. RT PCR was performed using SYBR Green assays (Applied Biosystems) and specific transcript levels were normalized to the housekeeping gene (GAPDH): CAA GGC TGA GAA CGG GAA G, GGT GGT GAA GAC GCC AGT. The following human primers were used: *RANK*: GCC TTG CCT GTA TCA CAA ACT, GCT GTA ACA AAT GTG AAC CAG GA^18^ *OSM*: TAA CAG CGG CAG GGT GTC, GAG AAA ACA GGT CCA CAG AGG T; *TNFRSF4*: GCA ATA GCT CGG ACG CAA TCT, GAG GGT CCC TGT GAG GTT CT; *TNFRSF18*: TTC AGT TTT GGC TTC CAG TGT, AGC GTT GTG GGT CTT GTT C and *CCL4L1*: AGG ACT CAC TGG GGT CAG C, CTT TTC TTA CAC CGC GAG GA. Primers were designed with Primer 3 software (http://biotools.umassmed.edu/bioapps/primer3_www.cgi)^19^ and purchased from IDT.

PCR was performed using the Applied Biosystems 7500HT Fast Real-PCR System and the ∆Ct calculation method was used to determine gene expression, as previously described.^20^

### Western blotting

RIPA buffer (Sigma) supplemented with protease and phosphatase cocktail inhibitor (1:100 – Thermo Scientific) was used for generating whole-cell lysates. Nuclear and cytoplasmic lysates were generated via a nuclear extract kit (Active Motif) according to the manufacturer’s instruction. Total protein was determined using a BCA protein assay kit (Thermoscientific). 10 μg or 50 μg of total protein from whole-cell lysates or nuclear/cytoplasmic lysates, respectively, were fractionated on a 4–12% Bis-Tris gel (Invitrogen) and transferred via iBlot (Invitrogen) to nitrocellulose membrane. Blocking was performed in 5% nonfat milk (0.05% Tween [Sigma] PBS). All antibodies were purchased from New England Biolabs and used at 1:1000 dilution. Chemiluminescence was detected using SuperSignal West Pico or West Femto substrate (Thermoscientific), and the Chemiluminescent Western Blot Scanner c-digit (*LI-COR*) or X-ray film. Image studio lite software or ImageJ analysis software was used to quantify band density.

### OSM ELISA

Cell-free supernatants from RANKL/RANKL & SIC exposed CD14+ monocytes derived from healthy individuals were measured via the Human Oncostatin M (OSM) or CCL24 duoset ELISA kits (R&D systems), according to manufacture’s instructions. The assay range was 31.30–2,000 pg/ml.

*Epigenetic analysis of the RANK gene locus via the EpiSwitch*^TM^ *3C assay*

Two million CD14^+^ monocytes were isolated from healthy donors or active MM patients and treated with M-CSF for 24 h, followed by treatment with RANKL for a further 24 h in the absence or presence of SIC. These cells were re-suspended in 1 ml of PBS, followed by the addition of 125 µl 32% PFA (4% PFA) and then rotated at room temperature for 25 mins to fix (cross-linking of interacting DNA) the cells. Fixation was quenched by adding 335 µl of 2 M Glycine to the cells, followed by rotation at room temperature for 5 mins. Samples were centrifuged at 5,000 g for 10 mins at 4°C. Supernatants were removed and cell pellets frozen at −80°C for subsequent evaluation via the EpiSwitch^TM^ assay. To perform the EpiSwitch^TM^ assay, chromatin from 0.5 × 10^6^ cells was extracted. Briefly, the higher order structures were fixed with formaldehyde, the chromatin extracted, digested with TaqI, diluted and ligated in conditions to maximize intramolecular ligation, and subsequent proteinase K treatment. Using proprietary EpiSwitch^TM^ software (Oxford Biodynamics, UK), potential chromatin interactions in the RANK loci were identified and primers designed.^21^ Primers were tested on templates to confirm activity. To accommodate for technical and replicate variations, each sample was processed four times. All the extracts from these replicates were pooled and the final nested PCR was performed on each sample. All PCR amplified samples were visualized by electrophoresis in the LabChip® GX from Perkin Elmer, using the LabChip DNA 1 K Version 2 kit (Perkin Elmer) and internal DNA marker was loaded on the DNA chip according to the manufacturer’s protocol using fluorescent dyes. Fluorescence was detected by laser and electropherogram read-outs translated into a simulated band-on-gel picture using the instrument software. The threshold of 30 fluorescence units and above was set to be positive.

### Mapping of CCS locations to genomic elements

EpiSwitch sites and data was loaded and visualized using the EpiSwitch Data Portal (EDP): https://episwitch3dgenomicsportal.com. The EP300 bed file was obtained from the ReMap database http://remap.univ-amu.fr/download_page.

### Transcription factor profiling arrays

The activity of 48 human transcription factors (TF) in 6 µg of nuclear lysate, created from human CD14+ monocytes, was determined utilizing the TF Activation Profiling Plate Array I, according to the manufacturer’s instructions (Signosis Inc.). The protocol has been described in detail previously.^22^ Log2 fold changes were first sorted by consistency across the replicates, then scaled to gene z-scores and heatmap was generated using the R package ggplot2.

### Statistics

Data were analyzed using Student’s t-tests or ANOVAs with post hoc tests as specified in the text. A p-value of ≤0.05 was considered significant. All analysis was carried out using GraphPad Prism software 9.3.1.

### Data availability

Micro array data: https://www.ncbi.nlm.nih.gov/geo/query/acc.cgi?acc=GSE133210

Code for Array data exploration and visualization:

https://github.com/Searchlight2

All other data supporting the findings of this study are available in the article and the supplementary information files, or from the corresponding author upon reasonable request.

## Results and discussion

*SIC inhibits multiple myeloma driven osteoclastogenesis in a dose dependent and irreversible manner, and reduces osteolytic function.*

**Figure 1.**
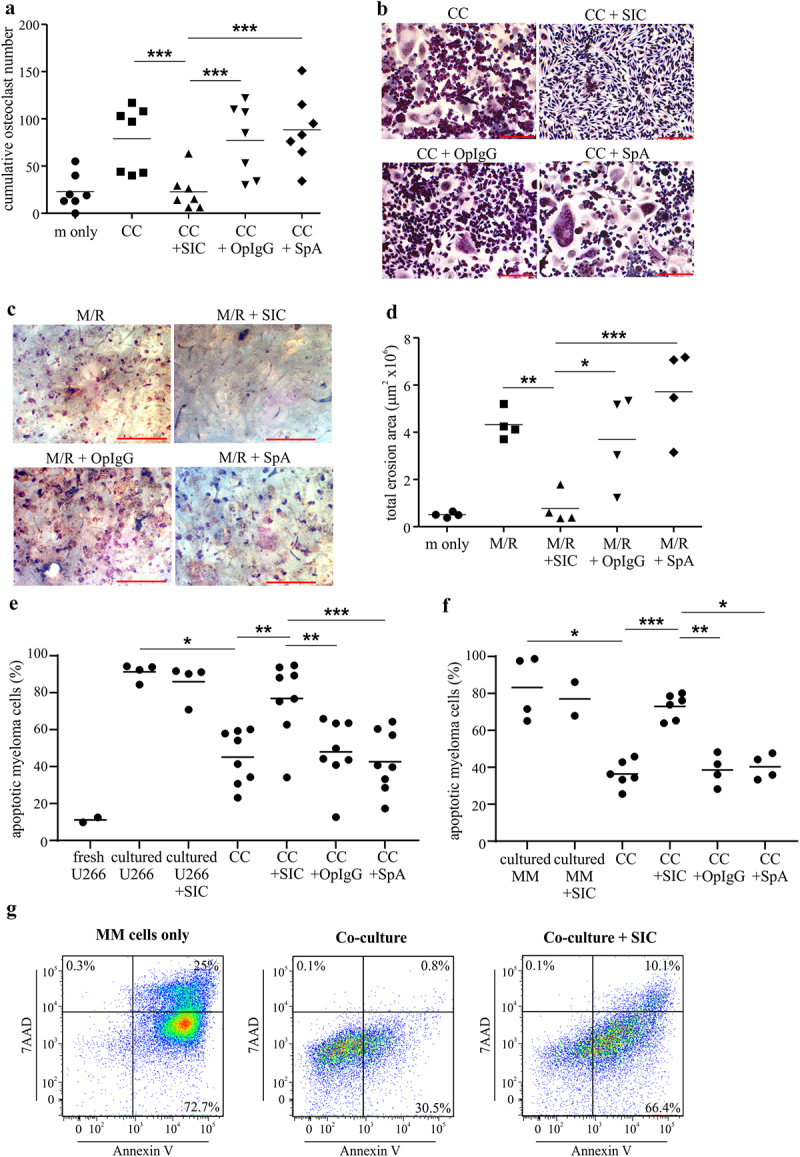
**SIC inhibits multiple myeloma induced osteoclastogenesis *in vitro* and increases myeloma cell apoptosis in co-culture with pre-osteoclasts** (a, b) Primary myeloma cells were isolated from bone marrow aspirates of active multiple myeloma (MM) patients using CD138+ magnetic selection. Myeloma cells were co-cultured (CC) with allogeneic CD14+ monocytes (pre-treated with M-CSF and RANKL for 2 days) in the presence or absence of SIC, OpIgG or SpA or with M-CSF only (m only) for 7 days. Cultures were performed in duplicates and cumulative osteoclast numbers from 8 random microscopic fields were determined by TRAP staining (≥ 3 nuclei and positive TRAP staining). (a) Shown are cumulative osteoclast numbers from 7 healthy donors’ monocytes, (one-way ANOVA with Tukey’s posttest). Representative TRAP stainings (**b**, scale bar: 200 µm) show marked reduction in MM cell induced osteoclastogenesis after SIC treatment. (c, d) CD14+ monocytes were cultured for 16 days with M-CSF and RANKL (M/R) on bone slices in the presence or absence of SIC, OpIgG or SpA or with M-CSF only (m only). Cultures were performed in triplicate, and the erosive potential was determined by lectin staining. (d) Analysis of total erosion area of 4 independent experiments shows significant reduction in osteoclast function after treatment with SIC (one-way ANOVA with Tukey’s posttest). Representative images of lectin staining are shown (**c**, scale bar: 500 µm). (e-g) Human U266B1 myeloma cells (e) or primary myeloma cells from active MM patients (f) were co-cultured (CC) with CD14+ monocytes (pre-treated with M-CSF and RANKL for 2 days) in the presence or absence of SIC, OpIgG or SpA. Myeloma cells were either analyzed directly (“fresh”), grown alone for 7 or 3 days (“cultured”) or in co-culture with pre-osteoclasts (“CC”). Myeloma cells were removed from the co-culture after 7 (**e**) or 3 days (f, g) and the percentage of apoptosis (Annexin V & 7AAD double positive in FACS) was analyzed. While co-culture with pre-osteoclasts had a positive effect on myeloma cell survival, the presence of SIC in the co-cultures significantly increased myeloma cell apoptosis (mixed effects analysis). (g) Representative FACS plots of primary MM cell apoptosis shown. *, p < .05; **, p < .01; ***, p < .001.

**Figure 2.**
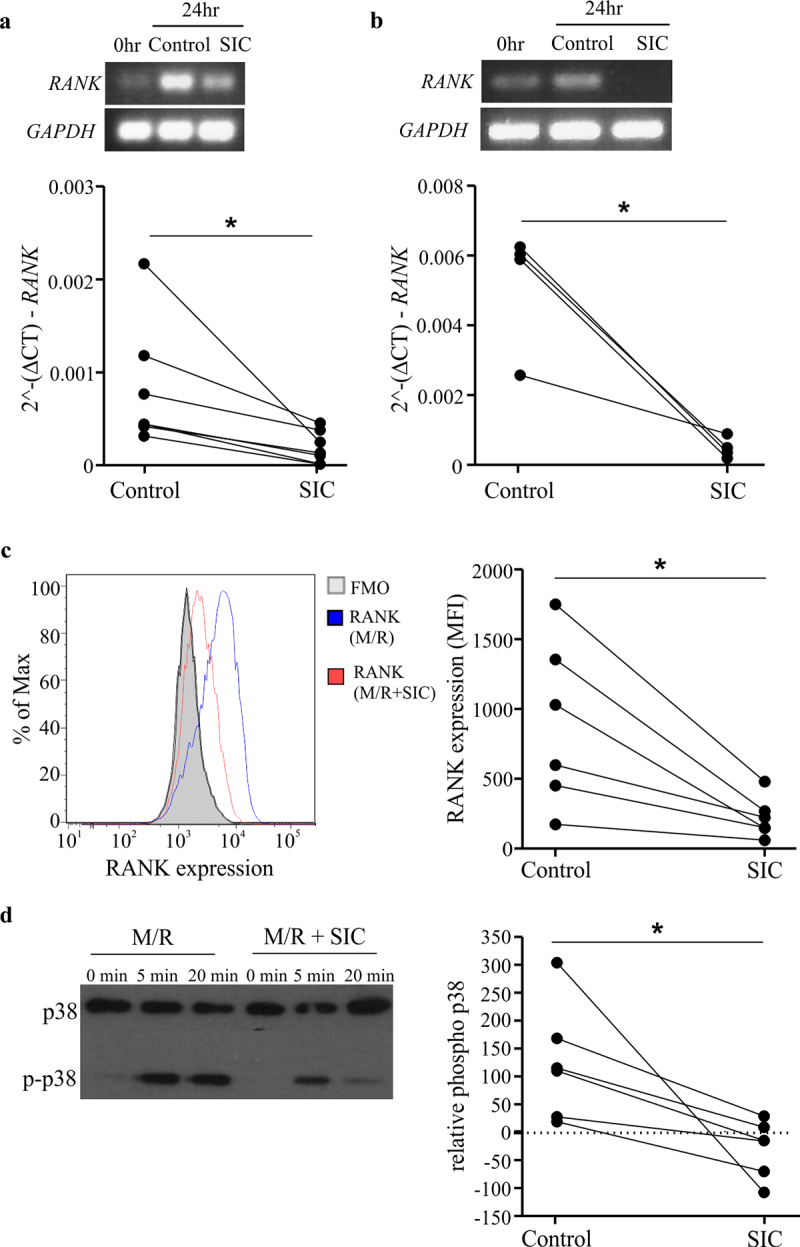
**SIC decreases RANK transcript and protein expression and reduces RANKL-induced signaling in pre-osteoclasts**. CD14+ monocytes derived from healthy individuals (**a**, n = 7) or active MM patients (**b**, n = 4) were cultured with M-CSF overnight, followed by incubation with RANKL for 24 h in the presence or absence of SIC. PCR was performed to assess the level of *RANK* mRNA, representative endpoint PCR and qPCR analysis (paired t-test) show marked reduction in *RANK* transcript levels after incubation with SIC. (c) CD14+ monocytes derived from healthy individuals were cultured with M-CSF and RANKL in the presence (red line) or absence of SIC (blue line). Cultures were performed in duplicates and after 2 days cells stained for RANK expression (binding of pacific blue labeled RANK). Levels of RANK protein were significantly reduced after treatment with SIC (paired t-test, n = 6). (d) CD14+ monocytes derived from healthy donors were treated with M-CSF and RANKL for 3 days in the presence or absence of SIC and serum starved for 6 h. After re-incubation with RANKL (100 ng/ml) for 5 or 20 min whole cell lysates were made and probed with antibodies against p38 and phosphorylated p38 MAPK. The percentage of phosphorylation was measured by densitometry and normalized to 0 min of each condition (n = 6). *, p < .05.

Prior studies have demonstrated that SIC, signaling through Fc**γ** receptor I (Fc**γ**RI), are capable of inhibiting osteoclastogenesis in the context of inflammation.^8^ To gain more insights into this Fc**γ**RI-dependent inhibition of bone erosion, we analyzed osteoclastogenesis in the prototypic non-inflammatory setting of multiple myeloma. Primary human MM cells were able to induce osteoclastogenesis *in vitro* independently of exogenous RANKL, as described previously^16,23^ and (Figure 1a). This MM cell-driven osteoclastogenesis was significantly inhibited after SIC treatment, both in an allogeneic (Figure 1**&b**) and syngeneic MM cell/pre-OC co-culture system (**Suppl**. Figure 1). Moreover, the inhibitory effect of SIC on RANKL-driven osteoclastogenesis was dose-dependent and irreversible (**Suppl**. Figure 2). Notably, SIC exposure significantly reduces the number of mature osteoclasts, and markedly decreases their osteolytic function (Figure 1c**&d**). In the MM bone marrow microenvironment, osteoclasts also enhance MM cell survival and growth, leading to a vicious circle of bone erosions and tumor growth. Evaluation of myeloma cell apoptosis in the MM cell/pre-OC co-culture system revealed that SIC not only drastically decreases osteoclast number and function, but also had a significant impact on myeloma cell survival (Figure 1e-1g).

### RANK gene and protein expression, and RANKL-mediated signaling is attenuated by SIC treatment

Since RANK-RANKL interactions are crucial for osteoclastogenesis, we first investigated the role of SIC on RANK expression and its downstream signaling. SIC treatment profoundly decreases *RANK* transcript levels in healthy (Figure 2a) and MM (Figure 2b) derived CD14^+^ monocytes. Monocytes also show reduced surface expression of RANK protein following SIC treatment (Figure 2c). This corresponded with a significant inability of RANKL to drive p38 MAPK signaling downstream of RANK (Figure 2d). Combined, this suggests that SIC inhibit osteoclastogenesis by down-regulating RANK transcript expression, which ultimately results in decreased RANKL/RANK signaling.

*SIC-modulates the transcriptional profile of CD14^+^ monocytes and drives them toward an anti-inflammatory/regulatory polarization state.*

**Figure 3.**
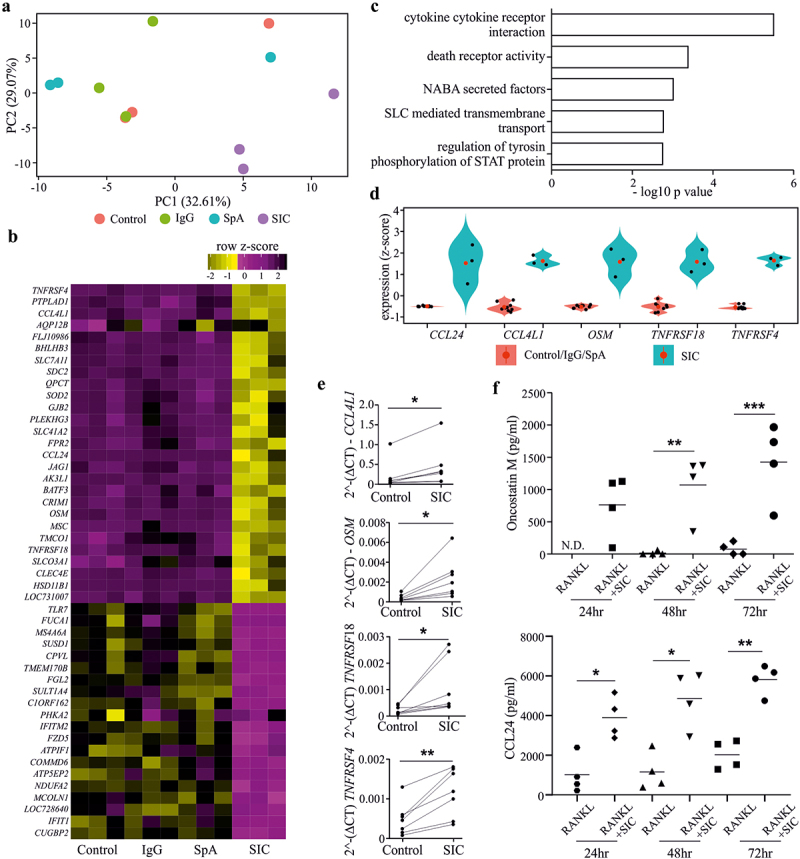
**Exposure of monocytes to SIC leads to differential expression of genes implicated in osteoclastogenesis**. CD14+ monocytes derived from healthy individuals (n = 3) were cultured with M-CSF overnight, followed by incubation with RANKL for 24 h in the presence or absence of SIC or the appropriate controls (IgG or SpA). Gene expression was determined by performing an Illumina HumanHT-12 V4.0 beadchip Array. The quantile normalized data was analyzed with Partek Genomic Suite. After log2 transformation a paired ANOVA was carried out on transcripts passing a low-expression cutoff filter (<6.1). Principal component analysis (PCA) (a), gene expression heat map (b) of all significant genes (FDR-adjusted p < .05) and gene ontology (GO) enrichment for all up-regulated genes (c, d) was performed with Searchlight. (**a**) Gene expression PCA shows PC1 (x-axis), PC2 (y-axis) and variance explained by each component; samples are colored by sample group. (**b**) Gene expression heatmap for the 47 significant genes (FDR adjusted p < .05) between control or IgG or SpA and SIC. Color intensity represents row scaled (z-score) expression level with yellow as high and pink as low. The y-axis has been hierarchically clustered using spearman values and mean agglomeration. SIC treatment results in a distinct expression profile (a, b), whereas IgG or SpA treatment did not induce any significant changes in the transcriptome compared to control cultures. (c) GO enrichment results (hypergeometric) for all up-regulated genes, showing the five most significant ontologies. (d) Gene expression boxplot for all significant genes in the “cytokine-cytokine receptor interaction” gene ontology. Expression levels are given as per gene z-scores. GO analysis for all up-regulated genes showed a significant enrichment for “cytokine-cytokine receptor interaction”. (e) qPCR validation of genes implicated in this pathway confirmed significant up-regulation of *Oncostatin M, TNFRSF4, TNFRSF18* and *CCL4L1* by SIC. 2^-(DCT) values (n = 7) are shown (paired t-test). (f) CD14+ monocytes were cultured with M-CSF overnight, followed by incubation with RANKL for 24, 48 and 72 h in the presence or absence of SIC. Oncostatin M and CCL24 protein levels in supernatants were determined by ELISA. Data from 4 independent experiments are shown (two-way ANOVA, Sidak’s posttest). *, p < .05; **, p < .01. N.D. = not detectable.

Given that SIC treatment down-regulates RANK transcript (and subsequent protein), which fundamentally stops cells responding to RANKL and thus differentiating down the osteoclast lineage, we wanted to understand the full extent of the early transcriptional changes that SIC induces in CD14^+^ monocytes. To achieve this, we performed a microarray study on RANKL and M-CSF treated CD14^+^ monocytes exposed to either SIC or appropriate controls (SpA or IgG alone) for 24 h. SIC treatment induced distinct transcriptional changes of 47 genes as compared to control; with 27 genes significantly up- and 20 genes down-regulated (FDR adjusted p-value < 0.05). In contrast, SpA or IgG treatment alone had no significant effect on gene transcription (Figure 3a&3B3). Gene ontology (GO) enrichment analysis revealed that genes up-regulated by SIC are significantly enriched for “cytokine–cytokine receptor interaction” (Figure 3c&3D3; Supplement Array data.xlsx). QPCR validation of *CCL4L1, Oncostatin M (OSM), TNFRSF18* and *TNFRSF4* confirmed that SIC treatment increases transcripts enriched for this GO term (Figure 3e). Moreover, this corresponded with increased secretion of OSM following SIC stimulation (figure 3f). In contrast, evaluation of CCL24 via QPCR did not corroborate the transcriptional change, however, we did observe a significant increase in the secretion of CCL24 following SIC stimulation (figure 3f). Notably, CCL24 has been associated with alternatively activated macrophages,^24^ which supports prior observations that SIC treatment can drive differentiation of macrophages with anti-inflammatory properties.^8^ Interestingly, OSM is widely implicated in bone turn-over,^25^ as it possesses the ability to drive the differentiation of mesenchymal stem cells into osteoblasts and thereby through the OSM-gp130-LIFR complex^26^ specifically promotes bone repair.^27^ Combined, this suggests that not only are SIC inhibiting cellular differentiation down the osteoclast lineage, but are potentially driving monocytes into an alternative/repair macrophage-like polarization state that have the potential to drive bone repair.^8^ It will be important in subsequent studies to determine the extent of the macrophage polarization state and bone repair characteristics of SIC-stimulated cells.

### SIC enhances STAT3 phosphorylation but decreases its ability to interact with DNA

**Figure 4.**
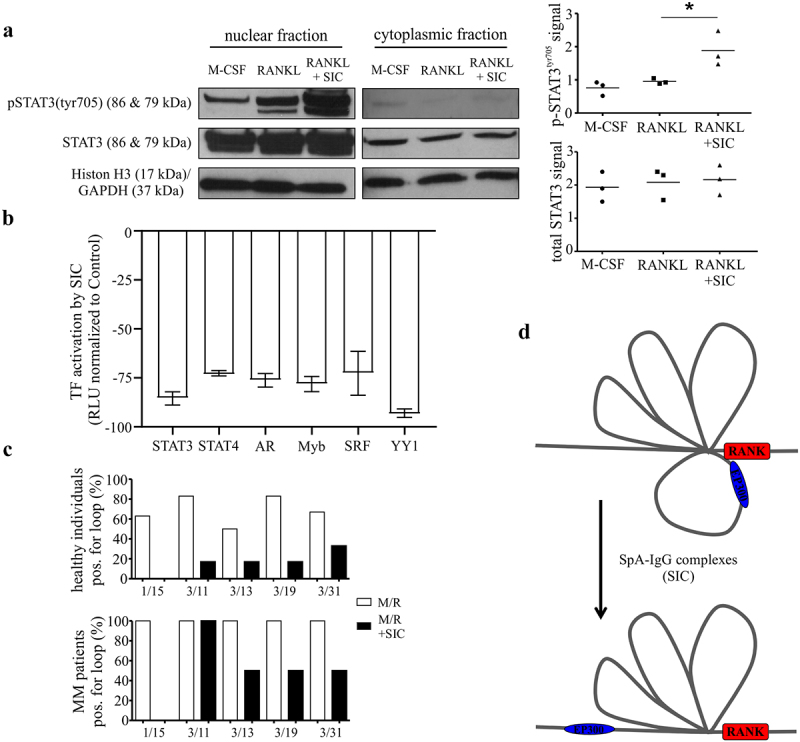
**SIC induces a decrease in transcription factor activation and the loss of specific chromosome loops at the RANK gene promoter**. (a, b) CD14+ monocytes (n = 3) from healthy individuals were cultured with M-CSF for 24 h followed by exposure to RANKL for a further 24 h in the presence or absence of SIC. Nuclear extracts were created and activation levels of transcription factors assessed by western blotting (**a**, phosphorylation of STAT3) or by Transcription Factor activation profiling arrays (Signosis, **b**). (a) Western blot band density was analyzed with ImageJ and densitometry values for phospho-STAT3 were normalized to total STAT3 densities (one-way ANOVA, Tukey’s posttest). (b) Shown is TF activation after SIC treatment for all TFs with a consistently reduced activity for all 3 monocyte sets (fold change < 2). Data is represented as relative light units (RLU) normalized to untreated monocytes (mean ± SEM). (c) CD14+ monocytes from healthy individuals (n = 6) and active MM patients (n = 2) were cultured with M-CSF for 24 h, followed by RANKL treatment for further 24 h in the absence or presence of SIC. Important epigenetic sites within and around the RANK promoter as previously identified by episwitch technology were analyzed by Oxford BioDynamics Ltd (OBD). Loop status analysis as determined by the 3C assay using different RANK primer pairs revealed a complete loss of chromosome looping between RANK primer 1 and 15 after treatment with SIC. (d) Schematic illustration of loss in formation of a specific spatially orientated chromosome loop at RANK promoter with the super enhancer EP300, following exposure to SIC. *, p < .05.

OSM has also been implicated in the “regulation of tyrosine phosphorylation of STAT protein”, another GO term enriched for genes up-regulated by SIC [Figure 3c and^[28,29]^]. Moreover, increased expression of p-STAT3^Tyr705^ has previously been associated with inhibition of OC differentiation.^30^ To investigate the relevance of this to the mechanism of SIC-mediated regulation of RANK expression, we examined the impact of SIC stimulation on p-STAT3^Tyr705^. Interestingly, SIC stimulation significantly increased the phosphorylation of nuclear STAT3 at residue Tyr^705^ (Figure 4a). However, evaluation of the ability of the SIC-activated STAT3, via Transcription Factor Activation Profiling Arrays, revealed a reduced capacity of activated STAT3 to bind to its corresponding consensus sequence (Figure 4b). This also corresponded with a reduced capacity of some additional transcription factors (TFs) to bind to their corresponding consensus sequences (Figure 4b), whilst most TFs were not consistently changed by SIC treatment (**Suppl**. Figure 3). These data therefore suggest that changes in STAT3 phosphorylation are not driving SIC-mediated inhibition of osteoclastogenesis.

### SIC-mediated downregulation of RANK is controlled by epigenetic changes in the RANK promoter

Having ruled out a potential role for STAT3 in SIC-mediated inhibition of osteoclastogenesis and the lack of any significantly enriched upstream regulators in the *in silico* analysis of the microarray, studies were initiated to investigate the possibility of SIC-inducing changes in chromosomal architecture. Importantly, chromosomal conformational loop formation (an essential mechanism for long-range chromatin interactions) can bring gene regulatory elements (e.g. enhancers or super-enhancers) into close proximity to gene promoter regions.^31^ To elucidate if SIC regulated *RANK* gene expression via changes at the level of three-dimensional chromosomal architecture, CD14^+^ monocytes from healthy individuals and MM patients were treated with M-CSF and RANKL. This was done in the absence or presence of SIC, followed by epigenetic analysis of the *RANK* promoter via a chromosome conformation capture (3C) EpiSwitch^TM^ assay.^32^ Screening of *in silico* defined chromosome loops via *RANK* loci primer pairs demonstrated that SIC treatment resulted in a consistent loss of one chromosome loop (1/15; chr18:59948993–59949322) in both healthy donor and MM monocytes (Figure 4c). Closer examination of the spatial orientation of this chromosomal loop and the associated genomic regions revealed that not only is this loop in a reverse direction compared to the other identified loops, but one anchor point is in close proximity to a p300 site (**Suppl**. Figure 4). Importantly, p300 sites have been shown to define genomic regions possessing super enhancer (SE) structure^33^ whilst the spatial orientation of 3-dimensional architecture has functional consequences on the ability of genomic regions to participate in activation of transcriptional machinery.^34–36^ These data therefore suggest that in an untreated scenario, the formation of loop 1/15 results in the p300 site being present in an optimal proximal position to enable enhanced transcription at the RANK loci (Figure 4d). In comparison, upon treatment with SIC this loop is lost and the 3-dimensional structure reverts to a state that is non-permissive for transcriptional activation (Figure 4d) and subsequent OC differentiation of the precursor cell.

Taken together, the results in this study provide novel insights into the molecular and epigenetic mechanisms by which Fc**γ**R-mediated downregulation of RANK, via SIC, is due to the loss of specific chromosome looping interaction at the *RANK* loci. Importantly, our study reveals new understanding of Fc**γ**R-mediated regulation of steady state and MM-driven osteoclastogenesis, and identifies unique molecular mechanisms, which could be exploited pharmacologically for the treatment of MM-associated bone disease. However, future studies should address how SIC-mediated FcγR signaling results in the epigenetic change at the RANK promoter, and experimentally confirm that the structural changes are influencing RANK transcript expression. Furthermore, although these studies have utilized a primary human cell *ex vivo* culture system, it is important that these findings are translated into a more clinically relevant context that emulates the complex marrow microenvironment observed in MM.

## Supplementary Material

Supplemental Material
