# Supplementary material for: Targeting 3D chromosomal architecture at the RANK loci to suppress myeloma-driven osteoclastogenesis
Source: Oncoimmunology. 2022 Aug 1;11(1):2104070. doi: 10.1080/2162402X.2022.2104070 (PMC9348127; doi:10.1080/2162402X.2022.2104070)
Supplement: Supplemental Material [file KONI_A_2104070_SM2503.zip › Supplemental Figure Captions.docx]

**Supplemental Figure Captions**

**Suppl. Fig. 1. SIC inhibits multiple myeloma induced osteoclastogenesis *in vitro*.** Primary myeloma cells swere isolated from bone marrow aspirates of active MM patients and co-cultured (CC) with syngeneic CD14+ monocytes (pre-treated with M-CSF and RANKL for 2 days) in the presence or absence of SIC, OpIgG or SpA or with M-CSF only (m only) for 7 days. SIC treatment significantly reduced cumulative osteoclast numbers in the co-culture as determined by TRAP staining (n=3, one-way ANOVA with Tukey’s post-test). *, p < 0.05

**Suppl. Fig. 2. SIC inhibits RANKL-driven osteoclastogenesis in a dose-dependent and irreversible manner.** (**A**) CD14+ monocytes were cultured for 7 days with M-CSF and RANKL in the presence or absence of SIC in different concentrations. Shown are cumulative numbers of osteoclasts of 4 independent experiments (TRAP staining, ≥2 nuclei). Significance was determined by one-way ANOVA with Tukey’s post-test. (**B**) CD14+ monocytes were cultured for 9 days with M-CSF and RANKL in the presence or absence of SpA, OpIgG or SIC and in one condition (open circles) SIC was removed after 4 days in culture. Cultures were performed in duplicates, cumulative osteoclast numbers from 8 random microscopic fields were determined by TRAP staining (≥ 3 nuclei) and data was analyzed with one-way ANOVA (Tukey’s post-test). SIC significantly inhibits osteoclastogenesis if present throughout the entire 9 days. Moreover, the same inhibitory effect is observed if SIC is only present for the first 4 days of culture (open circles). *, p < 0.05; ***, p < 0.001

**Suppl. Fig. 3. SIC induces decreased activation in a selection of transcription factors.**

CD14+ monocytes from healthy individuals were cultured with M-CSF for 24hrs followed by exposure to RANKL for a further 24hrs in the presence or absence of SIC. Nuclear extracts were created and activation levels of transcription factors assessed by Transcription Factor activation profiling arrays (Signosis). Shown is TF activation after SIC treatment for 3 monocyte sets. Data is represented as heatmap of log2 fold change for three replicates in each treatment group row-scaled into Z-scores with colour intensities representing a range of log2 fold changes (high in yellow, low in blue).

**Suppl. Fig. 4. Schematic of the RANK genomic region with associated EpiSwitch^TM^ chromosomal loops, anchor points, directionality and p300 sites.** The region on chromosome 18 from 59,916,913 to 60,068,301 with the EpiSwitch co-ordinates (Yellow and Green vertical lines), their orientation (F – forward, R- reverse) that form the chromosomal loop (Pink and Blue). The p300 sites in this region are indicated (Red vertical lines). The SIC effected loop (Pink) is indicated in the “RANK_loop_absent_anchor” line whilst the unaffected loops are in the “RANK_loop_present_anchor” line.
