# Supplementary figures and images for: Targeting 3D chromosomal architecture at the RANK loci to suppress myeloma-driven osteoclastogenesis
Source: Oncoimmunology. 2022 Aug 1;11(1):2104070. doi: 10.1080/2162402X.2022.2104070 (PMC9348127; doi:10.1080/2162402X.2022.2104070)

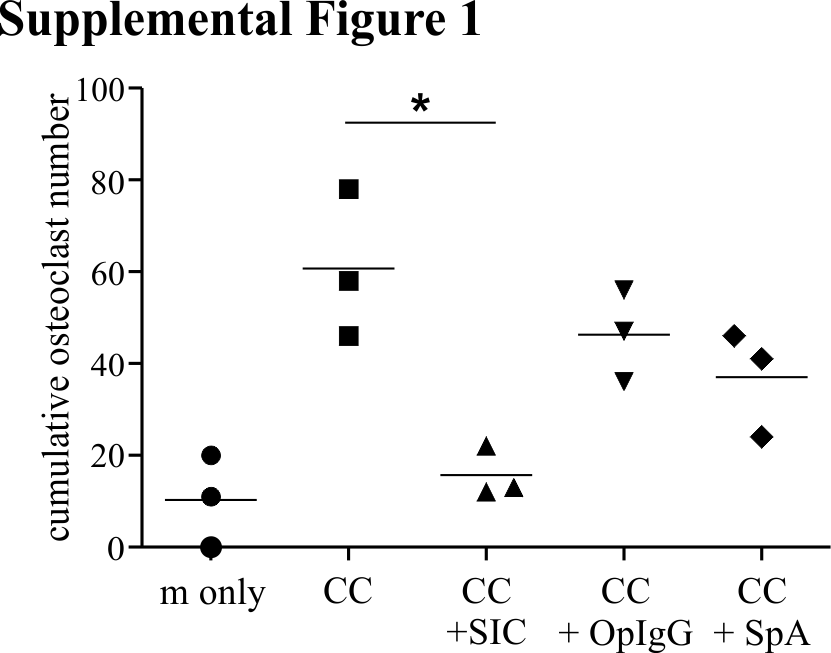

Supplement: Supplemental Material [file KONI_A_2104070_SM2503.zip › OI_Supplemental Figure 1.tif]

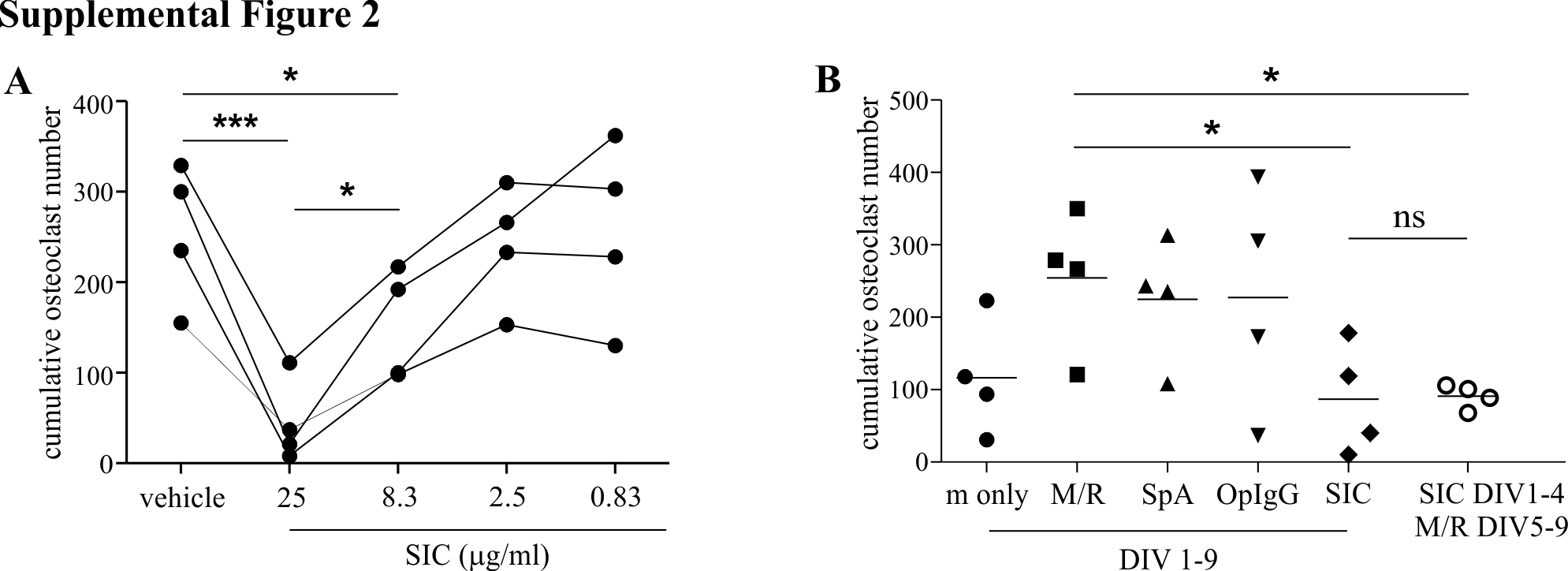

Supplement: Supplemental Material [file KONI_A_2104070_SM2503.zip › OI_Supplemental Figure 2.tif]

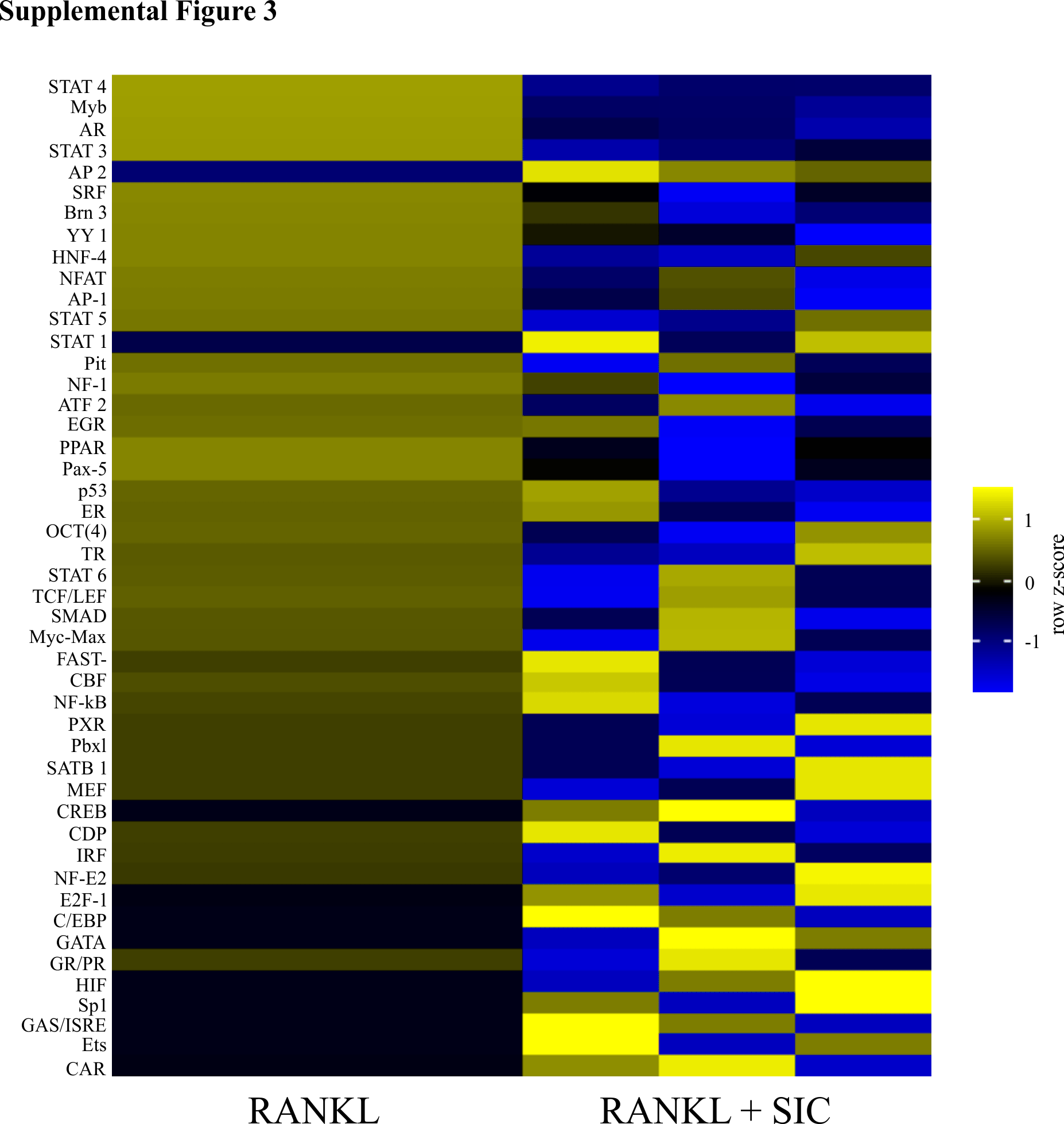

Supplement: Supplemental Material [file KONI_A_2104070_SM2503.zip › OI_Supplemental Figure 3.tif]

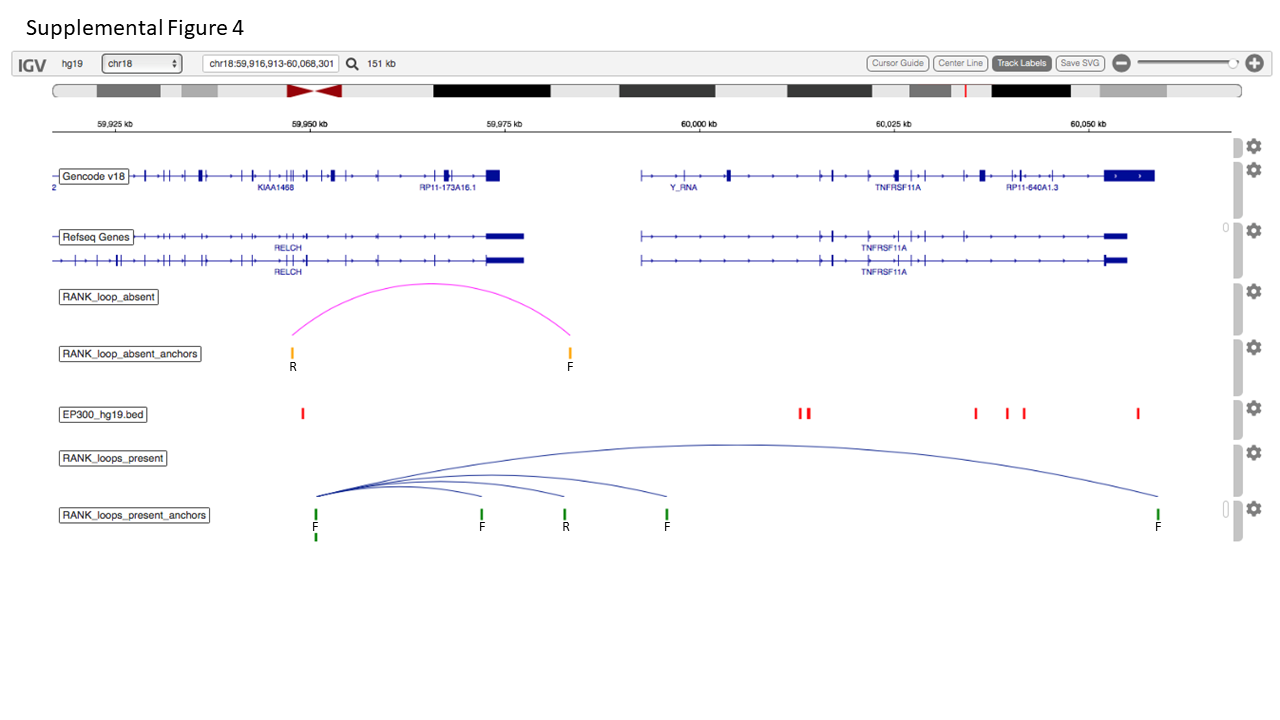

Supplement: Supplemental Material [file KONI_A_2104070_SM2503.zip › OI_Supplemental Figure 4.tif]
